# The Role of Metabolic Dysfunction–Associated Fatty Liver Disease in Developing Chronic Kidney Disease: Longitudinal Cohort Study

**DOI:** 10.2196/45050

**Published:** 2023-05-04

**Authors:** Suosu Wei, Jian Song, Yujie Xie, Junzhang Huang, Jianrong Yang

**Affiliations:** 1 Department of Scientific Cooperation of Guangxi Academy of Medical Sciences People’s Hospital of Guangxi Zhuang Autonomous Region Nanning China; 2 Institute of Cardiovascular Diseases of Guangxi Academy of Medical Sciences People’s Hospital of Guangxi Zhuang Autonomous Region Nanning China; 3 Department of Breast and Thyroid Surgery People’s Hospital of Guangxi Zhuang Autonomous Region Nanning China; 4 Department of Hepatobiliary, Pancreas and Spleen Surgery People’s Hospital of Guangxi Zhuang Autonomous Region Nanning China; 5 Institute of Health Management of Guangxi Academy of Medical Sciences People’s Hospital of Guangxi Zhuang Autonomous Region Nanning China

**Keywords:** chronic kidney disease, risk factor, kidney, renal, cohort study, metabolic dysfunction–associated fatty liver disease, MAFLD, incidence rate, incidence, liver, chronic disease, prevalence, association

## Abstract

**Background:**

The association between metabolic dysfunction–associated fatty liver disease (MAFLD) and chronic kidney disease (CKD) is unclear.

**Objective:**

This longitudinal cohort study aimed to test whether MAFLD plays an important role in the development of CKD.

**Methods:**

This cohort study included 41,246 participants who had undergone 3 or more health examinations from 2008 to 2015 at the People’s Hospital of Guangxi Zhuang Autonomous Region, China. Participants were categorized into 2 groups according to whether they presented with or without MAFLD. The occurrence of new-onset CKD was stated as either an estimated glomerular filtration rate of <60 mL/min per 1.73 m^2^ or a higher level of albuminuria during their follow-up appointment. The association between MAFLD and CKD was evaluated using a Cox regression method.

**Results:**

Of the 41,246 participants, 11,860 (28.8%) were diagnosed with MAFLD. Over the course of the 14-year follow-up (median 10.0 years), 5347 (13%) participants experienced a new incident of CKD (135.73 per 10,000 person-years). MAFLD was discovered as an important risk factor for new incidents of CKD (hazard ratio 1.18, 95% CI 1.11-1.26) by using the multivariable Cox proportional hazard regression model. When stratified by gender, the adjusted hazard ratio for the incidence of CKD in men and women with MAFLD were 1.16 (95% CI 1.07-1.26) and 1.32 (95% CI 1.18-1.48), respectively. According to the subgroup analysis results, after adjusting for confounding factors, the MAFLD-related CKD risk was greater in men aged <60 years (*P*_interaction_=.001) and in those with combined dyslipidemia (*P_interaction_*=.02), but this relationship was not found in women (all *P_interaction_*>.05).

**Conclusions:**

MAFLD plays an important role in the development of new incidents of CKD in the long run.

**Trial Registration:**

Chinese Clinical Trial Registry ChiCTR2200058543; https://www.chictr.org.cn/showproj.html?proj=153109

## Introduction

Nowadays, kidney disease poses a considerable burden on global health, and it affects global morbidity and mortality. In particular, there are about 10% of adults worldwide who experience chronic kidney disease (CKD) directly, which constitutes a big public health issues [1]. Global health policy makers should pay more attention to CKD, which is a preventable and treatable condition [1]. Currently, more than 2.5 million people receive kidney transplants, and this will reach 5.4 million by 2030. CKD can develop into end-stage renal disease, which can be fatal if renal replacement therapy is not an option [2]. The prevalence of CKD is 10.8% in China [3]. Therefore, it is of great public health importance to explore the related risk factors of CKD and take active preventive measures.

Nonalcoholic fatty liver disease (NAFLD) is characterized by hepatic steatosis without excessive alcohol consumption or other competing factors and fat accumulation in more than 5% of hepatocytes [4]. About 25% of adults worldwide currently have NAFLD [5]. Approximately 30% of Chinese adults have NAFLD, and rural areas are less likely to have cases than urban areas. In addition, men are more likely to have it than women [6]. Obesity, hypertension, and diabetes are metabolic risk factors for both NAFLD and CKD [7]. In patients who have NAFLD, the prevalence rate of CKD is 20% to 55%, and this is reduced to 5% to 35% for patients who do not have NAFLD. Some research results have found a correlation between NAFLD and CKD, which showed that NAFLD increases the risk of developing CKD on its own [8-12].

Due to the limitations in the definition for NAFLD in the clinical setting, in 2020, an international group of experts established the conception of metabolic dysfunction–associated fatty liver disease (MAFLD). This incorporated a diagnostic criteria of MAFLD that was based on fatty liver disease combined type 2 diabetes mellitus (T2DM) as well as either overweight or obesity or metabolic dysfunction [13,14]. The definition of MAFLD reflects that this type of liver disease is a series of complex metabolic disorders that do not rule out either excessive drinking or other chronic liver diseases [15,16]. It has been shown that patients with MAFLD have more metabolic complications than those with NAFLD and they have increased risks of advanced liver fibrosis and CKD [17,18]. However, due to several inconsistent reports regarding the correlation between MAFLD and CKD, this needs to be clarified. Therefore, we analyzed the relationship between MAFLD and new-onset CKD by conducting a 14-year retrospective cohort study.

## Methods

### Study Design and Participants

**Figure 1:**
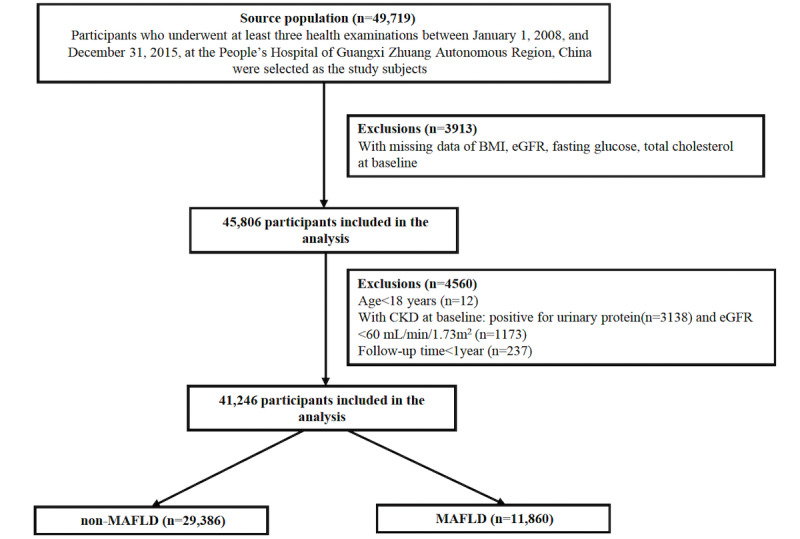
Flow chart of the selection of participants in the active health management platform. CKD: chronic kidney disease; eGFR: estimate glomerular filtration rate; MAFLD: metabolic dysfunction–associated fatty liver disease.

We used an active health management platform to construct a retrospective cohort study, and the protocol of study was registered on the Chinese Clinical Trial Registry (ChiCTR2200058543) [19]. Briefly, the active health management platform used an advanced medical data management system to manage patients and connected and indexed all the diagnostic and treatment records held at the hospital. It contained the outpatient, inpatient, and physical examination data with respect to the diagnosis of the patients. In addition, the treatment data, test reports, examination reports, electronic medical records, and other medical data of the outpatient, inpatient, and physical examinations were also recorded. All the medical information could be accessed from this platform, and when a patient comes to the clinic, the information is automatically integrated into this platform. This study enrolled participants who had received at least 3 health examinations and had an abdominal ultrasonography performed from 2008 to 2015 at the People’s Hospital of Guangxi Zhuang Autonomous Region, China (n=49,719). The date of the first health examination was defined as the index date. The exclusion criteria in this study were participants with (1) an estimated glomerular filtration rate (eGFR) <60 mL/min per 1.73 m^2^ or a high level of proteinuria at baseline, (2) missing either demographic or laboratory data, and (3) follow-up time <1 year. After exclusion, 41,246 participants were recruited into this study (Figure 1).

### Ethics Approval

The guidelines of the Declaration of Helsinki (6th revision, 2008) were followed in this study. The People’s Hospital of Guangxi Zhuang Autonomous Region Ethics Committee approved this study (KY-QT-202203). As the data were anonymized, individual informed consent was not needed for this study.

### Ascertainment of MAFLD

All enrolled participants were diagnosed with a fatty liver by hepatic ultrasound using the Asia-Pacific guidelines as a diagnostic guideline [20]. MAFLD was diagnosed if participants had a fatty liver and at least one the following criteria was met:

1. BMI ≥23 kg/m^2^
2. Diagnosed with T2DM
3. BMI <23 kg/m^2^ and 2 or more metabolic abnormalities for Asian men and women, including a waist circumference ≥90/80 cm, blood pressure ≥130/85 mmHg, plasma triglyceride (TG) ≥1.70 mmol/L, plasma high-density lipoprotein cholesterol (HDL-C) <1.0 mmol/L for men and <1.3 mmol/L for women, prediabetes (fasting glucose level of 5.6-6.9 mmol/L, 2-hour postload glucose level of 7.8-11.1 mmol/L, or hemoglobin A_1c_ of 5.7%-6.4%), homeostasis model assessment of insulin resistance score ≥2.5 (this study did not include these data), and a plasma high-sensitivity C-reactive protein level >2 mg/L (this study not include these data) [14].

### Measurements

Baseline data including sociodemographic, anthropometric, and clinical information and laboratory results were retrieved from the active health management platform. If any data were missing for the index date, then the data relating to the nearest index date were obtained. The indicator of renal function in this study was eGFR, which was based on the CKD Epidemiology Collaboration equation [21]. The covariates assessed in this study included BMI, T2DM, hypertension, and dyslipidemia. BMI was stratified into normal weight (<23 kg/m^2^) and overweight or obese (≥23 kg/m^2^). Participants with systolic blood pressure ≥140 mmHg or diastolic blood pressure ≥90 mmHg were considered to have hypertension. Participants with fasting blood glucose ≥7.0mmol/L or hemoglobin A_1c_ ≥ 6.5% were considered to have T2DM. Participants with total cholesterol ≥5.2 mmol/L, TG ≥1.7 mmol/L, low-density lipoprotein cholesterol (LDL-C) ≥3.4 mmol/L, or HDL-C <1.0mmol/L were considered to have dyslipidemia. All the laboratory tests were conducted within the health examination hospital.

### Study Outcome

All participants were followed prospectively until their last health examinations and medical visit in the period from 2016 to 2022. As a clinical end point, participants who have eGFR <60 mL/min per 1.73 m^2^ or 2 incidents of albuminuria were considered to be a new incident of CKD during the follow-up period.

### Statistical Analysis

For categorical variables, we presented counts and percentages, and for continuous variables, we presented medians and IQRs. For baseline data between participants with and without MAFLD, we used Mann-Whitney *U* and chi-square tests to compare continuous data and categorical data, respectively. The incidence of CKD was calculated by dividing the total number of newly diagnosed CKD cases by the total number of person-years contributed by participants during follow-up. We used Kaplan-Meier analysis to estimate the cumulative incidence of CKD between patients with and without MAFLD and log-rank tests to compare the differences between the 2 groups. We used Cox hazard models to calculation the hazard ratios (HRs) and 95% CIs for the risk between MAFLD and CKD. We used Schoenfeld residuals to evaluate the proportional hazards assumption. We selected covariates according to the reverse selection procedure and confounding factors reported in the previous literature [18]. Age and gender were adjusted in model 1. In addition, comorbidities of hypertension, overweight or obesity, dyslipidemia, and T2DM were adjusted in model 2. Furthermore, LDL-C, alanine aminotransferase (ALT), serum aspartate aminotransferase (AST), and serum creatinine were adjusted in model 3. Finally, stratified analysis was conducted in predesignated subgroups according to gender; age; overweight or obesity; and the presence of hypertension, dyslipidemia, and T2DM. We used R software packages (version 3.6.3; R Foundation for Statistical Computing) to conduct statistical analysis in this study. All test for *P* values were 2-tailed, and *P*<.05 was considered statistically significant.

## Results

### Baseline Characteristics

After applying the exclusion criteria, 41,246 participants were finally included for analysis in this cohort study. Among the included participants, 11,860 (28.8%) were diagnosed with MAFLD. These participants were more likely to be older; be men; have T2DM, hypertension, or dyslipidemia; and be either overweight or obese (all *P*<.001). The participants with MALFD also had higher BMI, fasting glucose, total cholesterol, LDL-C levels, and TG and lower HDL-C levels, gamma-glutamyl transferase, ALT, AST, and eGFR than participants without MAFLD (all *P*<.001; Table 1).

**Table 1:** Baseline characteristics of the participants in this study.

Characteristic	Total (N=41,246)			Men (n=22,417)				Women (n=18,829)		
	Non-MAFLD^a^ (n=29,386)	MAFLD (n=11,860)	*P* value	Non-MAFLD (n=13,355)	MAFLD (n=9062)	*P* value	Non- MAFLD (n=16,031)		MAFLD (n=2798)	*P* value
Age (years), median (IQR)	37.0 (29.0-46.0)	45.0 (37.0-53.0)	<.001	37.0 (29.0-47.0)	43.0 (35.0-50.0)	<.001	37.0 (29.0-45.0)		50.0 (43.0-57.0)	<.001
Gender, men, n (%)	13,355 (45.4)	9062 (76.4)	<.001	N/A^b^	N/A	N/A	N/A		N/A	N/A
BMI (kg/m^2^), median (IQR)	23.5 (21.6-25.4)	26.7 (25.0-28.6)	<.001	24.3 (22.6-26.1)	26.9 (25.3-28.7)	<.001	22.7 (21.0-24.5)		26.1 (24.4-28.4)	<.001
Waist circumference (cm), median (IQR)	83.0 (77.0-89.0)	93.0 (88.0-98.0)	<.001	87.0 (83.0-92.0)	94.0 (90.0-99.0)	<.001	79.0 (74.0-85.0)		87.0 (84.0-93.0)	<.001
Systolic blood pressure (mmHg), median (IQR)	116.0 (107.0-127.0)	129.0 (119.0-140.0)	<.001	122.0 (114.0-132.0)	129.0 (120.0-140.0)	<.001	111.0 (103.0-121.0)		128.0 (116.0-141.0)	<.001
Diastolic blood pressure (mmHg), median (IQR)	71.0 (65.0-79.0)	80.0 (73.0-87.0)	<.001	74.0 (68.0-82.0)	81.0 (74.0-88.0)	<.001	69.0 (62.0-76.0)		76.0 (70.0-85.0)	<.001
Diabetes mellitus, n (%)	1393 (4.7)	2234 (18.8)	<.001	831 (6.2)	1652 (18.2)	<.001	562 (3.5)		582 (20.8)	<.001
Hypertension, n (%)	2986 (10.2)	3665 (30.9)	<.001	1935 (14.5)	2822 (31.1)	<.001	1051 (6.6)		843 (30.1)	<.001
Dyslipidemia, n (%)	13,321 (45.3)	9110 (76.8)	<.001	7346 (55)	7228 (79.8)	<.001	5,975 (37.3%)		1882 (67.3)	<.001
Overweight or obesity, n (%)	16,699 (56.8)	11,362 (95.8)	<.001	9382 (70.3)	8807 (97.2)	<.001	7317 (45.6)		2555 (91.3)	<.001
Fasting glucose (mmol/L), median (IQR)	5.0 (4.6-5.3)	5.3 (4.9-5.8)	<.001	5.0 (4.7-5.4)	5.3 (4.9-5.8)	<.001	5.0 (4.6-5.3)		5.4 (5.0-5.9)	<.001
ALT^c^ (unit/L), median (IQR)	16.0 (12.0-23.0)	26.0 (19.0-38.0)	<.001	20.0 (15.0-28.0)	29.0 (21.0-41.0)	<.001	14.0 (11.0-19.0)		20.0 (15.0-27.0)	<.001
AST^d^ (unit/L), median (IQR)	20.0 (17.0-24.0)	23.0 (20.0-28.0)	<.001	22.0 (18.0-26.0)	24.0 (20.0-29.0)	<.001	19.0 (16.0-22.0)		21.0 (18.0-25.0)	<.001
GGT^e^ (unit/L), median (IQR)	18.0 (13.0-27.0)	34.0 (23.0-54.0)	<.001	25.0 (18.0-37.0)	38.0 (26.0-60.0)	<.001	14.0 (11.0-18.0)		21.0 (16.0-29.0)	<.001
TC^f^ (mmol/L), median (IQR)	4.8 (4.2-5.4)	5.3 (4.7-5.9)	<.001	4.8 (4.3-5.5)	5.3 (4.7-5.9)	<.001	4.7 (4.2-5.3)		5.3 (4.7-6.0)	<.001
HDL^g^ cholesterol (mmol/L), median (IQR)	1.4 (1.2-1.7)	1.2 (1.0-1.4)	<.001	1.3 (1.1-1.5)	1.1 (1.0-1.3)	<.001	1.5 (1.3-1.8)		1.3 (1.1-1.5)	<.001
LDL^h^ cholesterol (mmol/L), median (IQR)	3.0 (2.5-3.5)	3.4 (3.0-4.0)	<.001	3.2 (2.6-3.6)	3.4 (3.0-4.0)	<.001	2.9 (2.4-3.4)		3.4 (3.0-4.0)	<.001
TG^i^ (mmol/L), median (IQR)	1.0 (0.7-1.4)	1.8 (1.3-2.7)	<.001	1.1 (0.8-1.6)	1.9 (1.3-2.8)	<.001	0.8 (0.6-1.1)		1.5 (1.1-2.2)	<.001
Creatinine (μmol/L), median (IQR)	70.0 (59.0-84.0)	80.0 (69.0-90.0)	<.001	85.0 (78.0-93.0)	84.0 (77.0-92.0)	<.001	60.0 (55.0-66.0)		61.0 (55.0-67.8)	<.001
eGFR^j^ (mL/min per 1.73 m^2^), median (IQR)	104.7 (92.5-115.5)	97.0 (86.1-106.6)	<.001	98.3 (87.4-109.1)	96.2 (85.4-106.1)	<.001	109.7 (98.7-119.1)		99.8 (88.8-108.2)	<.001

^a^MAFLD: metabolic dysfunction–associated fatty liver disease.

^b^N/A: not applicable.

^c^ALT: alanine aminotransferase.

^d^AST: aspartate transaminase.

^e^GGT: gamma-glutamyl transferase.

^f^TC: total cholesterol.

^g^HDL: high-density lipoprotein.

^h^LDL: low-density lipoprotein.

^i^TG: triglyceride.

^j^eGFR: estimated glomerular filtration rate.

### Incidence of CKD Between 14-year Follow-up

**Figure 2:**
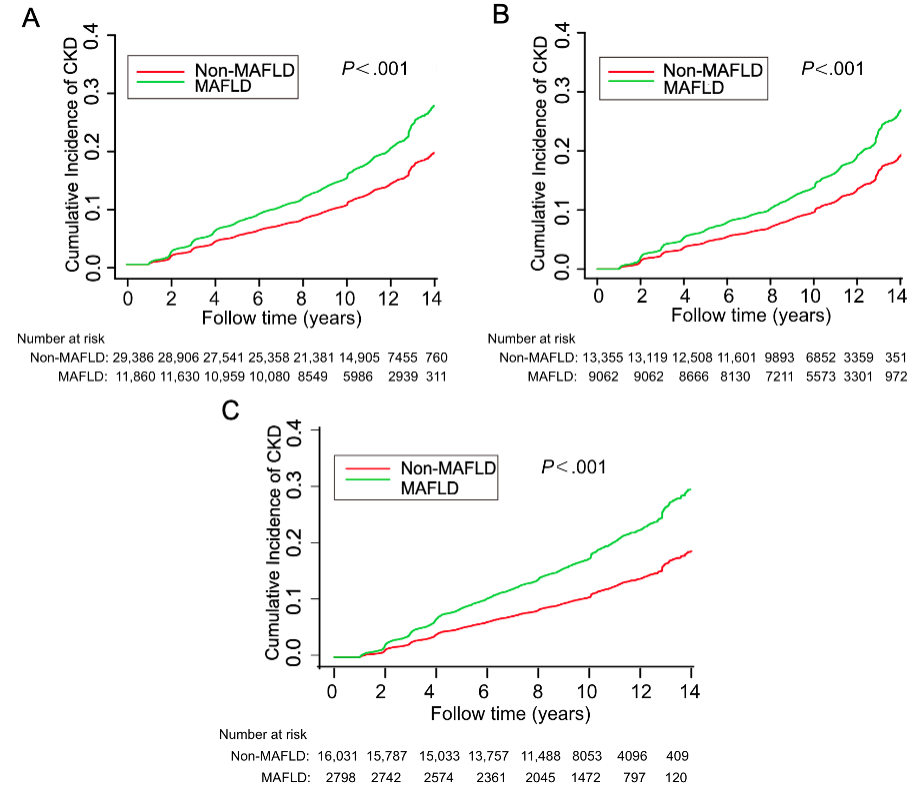
The cumulative incidence of chronic kidney disease (CDK) based on (A) the total number of participants, (B) men, and (C) women. MAFLD: metabolic dysfunction–associated fatty liver disease.

The median follow-up of this cohort study was 10.0 (IQR 7.7-12.0) years. During 393,933.1 person-years of follow-up, 5347 (13%) of the 41,246 participants developed new incidents of CKD (135.73 per 10,000 person-years, 95% CI 132.1-139.4). In the MAFLD group, the rate of new incidents of CKD was 177.9 (95% CI 170.2-185.8) per 10,000 person-years, whereas in non-MAFLD group, the rate of new incidents of CKD was 118.8 (95% CI 114.9-122.9) per 10,000 person-years. When stratified by gender, in the men, the new incidence of CKD was 167.0 (95% CI 158.4-175.8) per 10,000 person-years in the MAFLD group and 114.6 (95% CI 108.8-120.6) per 10,000 person-years in non-MAFLD group. In women, the new incidence of CKD was 212.5 (95% CI 195.5-230.6) per 10,000 person-years in the MAFLD group and 122.4 (95% CI 116.9-128.1) per 10,000 person-years in non-MAFLD group (Table 2). Kaplan-Meier analysis indicated that the cumulative incidence rate of CKD in the MAFLD group was significantly higher than that in the non-MAFLD group (*P*<.001). This result was similar to observed when the participants were stratified by gender (Figure 2).

**Table 2:** The incidence chronic kidney disease in participants with and without metabolic dysfunction–associated fatty liver disease.

Group		Events, n	Person-years	Incidence (per 10,000 person-years; 95% CI)	Hazard ratio (95% CI)					
					Model 1^a^	*P* value	Model 2^b^	*P* value	Model 3^c^	*P* value
**Total**										
	Non-MAFLD^d^	3342	281,207.0	118.8 (114.9-122.9)	Reference		Reference		Reference	
	MAFLD	2005	112,726.1	177.9 (170.2-185.8)	1.27 (1.19-1.34)	<.001	1.16 (1.09-1.24)	<.001	1.18 (1.11-1.26)	<.001
**Men**										
	Non-MAFLD	1473	128,539.8	114.6 (108.8-120.6)	Reference		Reference		Reference	
	MAFLD	1431	85,715.8	167.0 (158.4-175.8)	1.28 (1.19-1.38)	<.001	1.14 (1.05-1.24)	<.001	1.16 (1.07-1.26)	<.001
**Woman**										
	Non-MAFLD	1869	152,667.2	122.4 (116.9-128.1)	Reference		Reference		Reference	
	MAFLD	574	27,010.3	212.5 (195.5-230.6)	1.42 (1.28-1.57)	<.001	1.30 (1.16-1.45)	<.001	1.32 (1.18-1.48)	<.001

^a^Model 1: The total group was adjusted for age and gender. Men and woman groups were adjusted for age.

^b^Model 2: Model 1 with additional adjustments for hypertension, dyslipidemia, overweight or obesity, and diabetes mellitus.

^c^Model 3: Model 2 with additional adjustments for low-density lipoprotein, aspartate aminotransferase, alanine aminotransferase, and creatinine.

^d^MAFLD: metabolic dysfunction–associated fatty liver disease.

### MAFLD Is a Risk Factor in the Development of New Incidence of CKD

When age and gender were adjusted using multivariable Cox proportional hazard model, the result showed that the participants with MAFLD had a 1.27-fold higher HR for the incidence of CKD than those without MAFLD (adjusted HR 1.27, 95% CI 1.19-1.34). Even after the additional adjustments for hypertension, dyslipidemia, overweight or obesity, T2DM, LDL, AST, ALT, and creatinine, participants with MAFLD still had a higher risk in the development of new incidents of CKD (adjusted HR 1.18, 95% CI 1.11-1.26). When stratified by gender and compared with those without MAFLD, based on the fully adjusted Cox proportional hazard model, in men, the HR for the incidence of CKD in participants with MAFLD was 1.16 (95% CI 1.07-1.26), and in women, it was 1.32 (95% CI 1.18-1.48; Table 2).

### Subgroup Analysis

**Figure 3:**
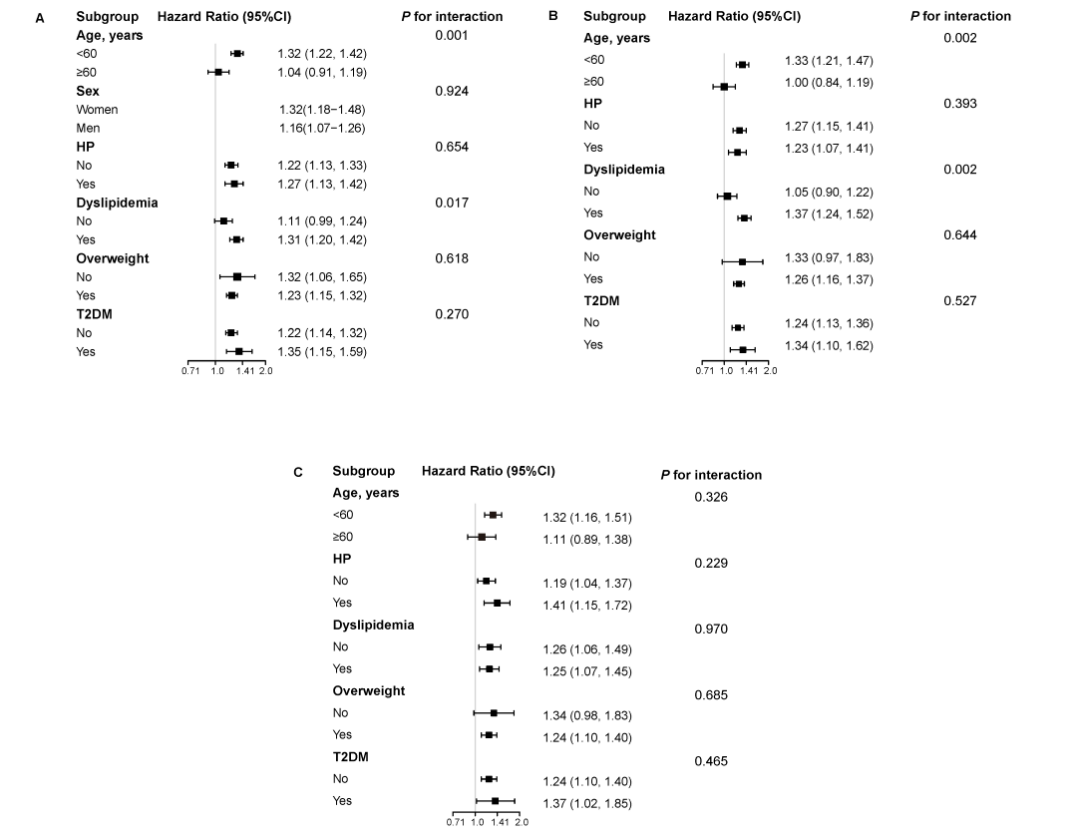
Forest plots showing the effect of metabolic-dysfunction associated fatty liver disease on the incidence of chronic kidney disease based on (A) the total number of participants, (B) men, and (C) women. Each stratification was adjusted for all the factors (age, gender, hypertension, dyslipidemia, overweight or obesity, type 2 diabetes mellitus [T2DM], low-density lipoprotein, aspartate aminotransferase, alanine aminotransferase, and creatinine) except the stratification factor itself.

In the subgroup analyses, we observed that in the total and men groups, the MAFLD-related CKD risk was stronger in patients aged <60 years and in those with combined dyslipidemia (*P_interaction_*=.001; Figure 3A-B), but this was not seen in the women group (*P_interaction_*=.02; Figure 3C). However, the other subgroup factors did not show any significant interaction discrepancies (all *P*>.05; Figure 3).

## Discussion

### Principal Findings

In this large, 14-year longitudinal cohort study, we found that participants with MAFLD have a higher risk for developing CKD than those without MAFLD. This was similar to the results found when the patients were stratified by gender. After adjusting for confounding factors such as demographics, laboratory parameters, and complications, these findings remained unchanged. Interestingly, we found that the MAFLD-related CKD risk was stronger in men aged <60 years and men with combined dyslipidemia but not in women.

This study provides further evidence to support the observations from several other studies regarding the relationship between MAFLD and CKD [18,22-24]. A cohort study with a median of 5.1 years of follow-up showed that participants with MAFLD had a 39% higher risk of new-onset CKD when compared to participants without MAFLD [18]. A cohort study with a median of 6.3 years of follow-up using a health examination database from a Japanese population similarly reported that compared to participants without MAFLD, participants with MAFLD had a 12% higher risk of new-onset CKD [22]. A cohort study with a median of 4.6 years of follow-up from China showed that participants with MAFLD had a 64% higher risk of incidents of CKD than those without MAFLD [24]. Another retrospective cohort study with a median of 4.6 years of follow-up from Japan also showed that those with MAFLD carried a 24% higher risk of incidents for CKD [23]. The results of this 14-year longitudinal study found that participants with MAFLD had a 18% higher risk of incidents of CKD when compared to those without MAFLD. For men and women, the risk of incidents for CKD changed to 16% and 32%, respectively. These findings are consistent with the aforementioned studies. In this study, as shown in a further subgroup analysis when metabolic factors were adjusted, the relationship between MAFLD and CKD remained unchanged. This may be due to the definition of MAFLD, which focuses more on the metabolic aspects of the disease.

The development of CKD can cause irreversible decline of kidney function, and this will lead to a need for dialysis and an increase in the number of deaths [1]. However, studies have shown that the epidemiology of CKD varies according to gender, and more women than men were affected by this disease, especially when stage G3 CKD is taken into consideration [25]. Therefore, when discussing the relationship between MAFLD and CKD, the data should be considered separately according to gender. The reason why women have a higher incidence of CKD may be related to the overdiagnosis of CKD by using the eGFR equation. It may also reflect the longer life expectancy observed in women [25,26]. Our results show that men have a higher prevalence of MAFLD, however, women have a higher risk of incidents of CKD. In addition, this study showed that men aged <60 years and men with combined dyslipidemia had a particular higher risk of CKD. However, this phenomenon has not been observed in women.

The gender difference observed may be due to the fact that there are some variables in men that may contribute to obesity, diabetes, and metabolic syndrome that are different in women. A previous study had shown that different lifestyle factors such as a high lipid diet and smoking are potential factors for gender differences in the progression of CKD [25]. On the other hand, this may be related to the lower age of men and higher age of women in the participants with MAFLD in this study. In addition, this study shown that the prevalence of dyslipidemia in men with MAFLD was also higher than that in women. Dyslipidemia can lead to inflammation, oxidative stress, and lipid toxicity, resulting in an impaired glomerular filtration barrier and proteinuria. The mechanism by which lipids can affect eGFR may be that a fatty liver damages the kidney through excessive very low–density lipoprotein secretion, which would induce atherosclerotic dyslipidemia [27]. The increased levels of circulating TG-rich lipoproteins and oxidized LDL-C can promote glomerular damage and mesangial cell proliferation. However, the potential mechanism of gender differences in the risk of CKD onset with dyslipidemia needs further study. This study shows the importance for the future development of more gender-specific interventions in medical practice. In particular, interventions should be given as early as possible, which might reduce the risk of incidents of CKD in men with MAFLD, especially in younger men with comorbid dyslipidemia. It is suggested that the renal function of patients with MAFLD should be evaluated regularly in clinical practice. A multidisciplinary and person-centered approach can be taken to manage patients with both MAFLD and CKD, as most of these patients have common metabolic comorbidities such as obesity, hypertension, atherosclerotic dyslipidemia, or T2DM. In addition, some drugs that target metabolic risk factors, such as glucagon-like peptide-1 receptor agonists and sodium-glucose cotransporter-2 inhibitors, can be given on a case-by-case basis and may benefit both the liver and kidneys in patients with MAFLD and CKD.

According to the latest definition, metabolic disorders are a key feature of MAFLD. Therefore, when compared with patients with NAFLD, those with MAFLD have a greater likelihood of complications in metabolism-associated diseases, but they are also more prone to liver fibrosis [17,28]. Since fibrosis-associated NAFLD correlates with CKD [29], the relationship between MAFLD and CKD may be closer than that with NAFLD. At present, there are only a few studies on the pathophysiological mechanism of MAFLD and CKD, but the factors that lead to kidney-liver crosstalk can be better explained than those in the pathogenesis that occurs between NAFLD and CKD. These may include genetic, internal environmental risk, and metabolic factors. First, many studies have shown that polymorphisms in genes such as *PNPLA3*, *HSD17B13*, *TM6SF2*, *MBOAT7*, and *GCKR* might play the important roles in the occurrence and development of NAFLD and CKD [17]. Second, a study showed that microbiota from the intestines as well as the intestinal barrier integrity might affect the correlation between NAFLD and CKD through the gut-liver-kidney signaling axis [30]. Studies have shown that the dysregulation of adipokines in patients with MAFLD, including decreased adiponectin and increased fatty acid–binding protein 4 [31], may lead to renal dysfunction [32], glomerular injury [33], tubule-interstitial injury [34], and a decline of eGFR [35]. Finally, the definition of MAFLD includes metabolic risk factors, such as obesity, hypertension, diabetes, and dyslipidemia, that have also been linked to increasing the risk of CKD incidents [36,37]. A study showed that, compared with participants without metabolic syndrome, participants with metabolic syndrome have a 2.5-fold higher risk of developing CKD [38]. Therefore, metabolic dysfunction may play a crucial role in the connection between MAFLD and CKD.

### Limitations

The highlight of this study is that it consisted of a large cohort of participants and had a long-term follow-up to observe the incidence of CKD. However, there are some limitations to this study. (1) The participants inhabited urban districts who received health examinations yearly in 1 hospital, and we excluded 1927 participants who had no data regarding MAFLD; therefore, the potential for selection bias was unavoidable. (2) Liver biopsies is the optimum way to diagnose fatty liver disease. However, this is an invasive method and ultrasonography was used instead to diagnose fatty liver disease in our health examinations. A study had shown that the sensitivity and specificity of ultrasonography were high for the diagnosis of fatty liver [39], and it is frequently used in large-scale epidemiological studies [40]. (3) Although 2 incidents of albuminuria were used as the clinical end point in this study, as kidney function was evaluated through an annual health examination, only qualitative data could be obtained. Therefore, at the end of the study, some of the participants may have had acute kidney injury. (4) The health examinations used in this study did not routinely measure either insulin or high-sensitivity C-reactive protein levels. Therefore, it is possible that MAFLD may have been misclassified in some participants. (5) Data relating to the pathological severity of hepatic steatosis were not collected in this study. (6) This study has a long follow-up period, and the metabolic status of participants may have changed over time. Therefore, this study only analyzes the metabolic status at baseline, which may not accurately reflect the true metabolic status of participants. (7) This study was not able to adequately collect participants’ smoking, drinking, and eating habits and concomitant therapy, such as the use of angiotensin-converting enzyme inhibitors or sartans, diuretics, statins, and antidiabetic drugs; therefore, the impact of living habits and concomitant therapy on outcomes could not be assessed. In addition, changes in baseline characteristics of the participants at follow-up were not included in the statistical analysis; therefore, the impact of changes in baseline characteristics over time on outcomes could not be assessed either. (8) The results of this study come from a population in China who underwent annual health examinations. It is likely that different ethnic groups are associated with different metabolic risk factors and further studies on a range of ethnic populations are required to verify this study.

### Conclusions

This retrospective cohort study found that there was a modest and independent correlation between MAFLD and CKD. It is necessary to explore the relationship between MAFLD and CKD through a large-scale cohort study and basic research and to develop new intervention strategies so that we can prevent the future occurrence of CKD in the population with MAFLD.

## Abbreviations

ALT: alanine aminotransferase
AST: aspartate aminotransferase
CKD: chronic kidney disease
eGFR: estimated glomerular filtration rate
HDL-C: high-density lipoprotein cholesterol
HR: hazard ratio
LDL-C: low-density lipoprotein cholesterol
MAFLD: metabolic dysfunction–associated fatty liver disease
NAFLD: nonalcoholic fatty liver disease
TG: triglyceride
T2DM: type 2 diabetes mellitus

## Data Availability

The data sets generated and analyzed during this study are not publicly available due to the inclusion of protected health information but can be made available after deidentification upon reasonable request to the corresponding author (JY).
